# The mutual regulatory loop between TPTEP1 and miR-1303 in leukemogenesis of acute myeloid leukemia

**DOI:** 10.1186/s12935-021-01966-0

**Published:** 2021-05-13

**Authors:** Li Li, Weidong Zhao

**Affiliations:** 1 Department of Lymphoma, Sichuan Cancer Hospital & Institute, Sichun Cancer Center, School of Medicine, University of Electronic Science and Technology of China, No.55, Section 4, South Renmin Road, Chendu, 610041 Sichuan China; 2 Food Nutrition Center, West China Hospital, Sichun University, No.37, Guoxue Xiang, Wuhou District, Chendu, 610041 Sichuan China

**Keywords:** AML, Leukemogenesis, TPTEP1, MiR-1303, JNK/c-JUN

## Abstract

**Background:**

Non-coding RNAs (ncRNAs) have been identified as key regulators during the pathogenesis and development of cancers. However, most of ncRNAs have never been explored in acute myeloid leukemia (AML).

**Methods:**

Gene expression was evaluated by quantitative real-time polymerase chain reaction (qRT-PCR) or western blot. Functional assays were performed to assess the cellular processes in AML cells. The relationship between genes was verified by means of a series of mechanism assays.

**Results:**

Transmembrane phosphatase with tensin homology pseudogene 1 (TPTEP1) was notably downregulated in AML cells, and functionally acted as a proliferation-inhibitor. Additionally, TPTEP1 suppressed AML cell growth by inactivating c-Jun N-terminal kinase (JNK)/c-JUN signaling pathway. MicroRNA (MiR)-1303, as an oncogene, was predicted and validated as a target of c-JUN in AML cells. Also, TPTEP1 interacted with miR-1303 and they were mutually silenced by each other in AML cells. Furthermore, the effect of TPTEP1 overexpression on AML cell proliferation was counteracted under miR-1303 upregulation.

**Conclusion:**

Our findings unmasked a feedback loop of TPTEP1/JNK/c-JUN/miR-1303 axis in AML cells, suggesting TPTEP1 and miR-1303 as potential targets for developing therapeutic strategies for AML patients. 
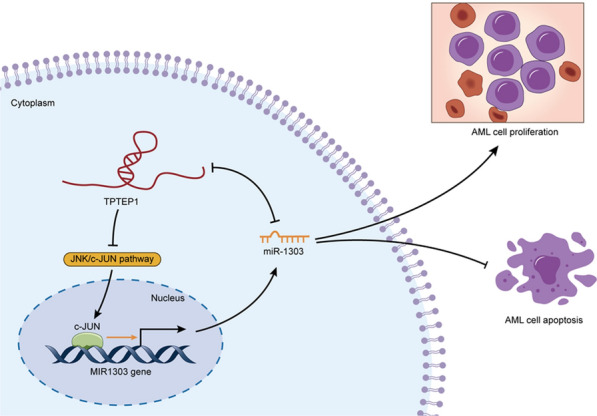

## Background

Acute myeloid leukemia (AML) is a clonal neoplastic disease featured with a growth in the amount of myeloid cells in the bone marrow of patients and arrest in cell maturation, and eventually leads to hematopoietic insufficiency [1, 2]. Recently, increasing reports have uncovered the increase of aberrant myeloid blasts along with serious blockage in myeloid differentiation are the primary signs of AML [3, 4]. China has the third highest incidence of leukemia in the world, and it mainly affects young people [5]. Also, the overall survival of AML patients is still disappointing although undergoing therapies [6]. Therefore, studies to probe new targets for the development of effective treatment for AML patients are badly needed.

In the past decades, long non-coding RNAs (lncRNAs) have been largely studied in human cancers [7]. Recently, the dysregulation and participation of lncRNAs in the formation and developmemt of a variety of cancers have been revealed [8], including AML [9–11]. As an example, ANRIL modulates AML progression through targeting AdipoR1/AMPK/SIRT1 pathway [12]. Transmembrane phosphatase with tensin homology pseudogene 1 (TPTEP1) is a novel lncRNA that has been previously unveiled to repress tumor progression in hepatocellular carcinoma by hindering Signal transducer and activator of transcription 3 (STAT3) phosphorylation [13]. Meanwhile, microRNAs (miRNAs) have been abundantly explored in human cancers, such as anti-miR-203 restricts estrogen receptor (ER)-positive breast cancer growth and stemness via modulating SOCS3[14], and the emerging role of miRNAs has also been reported in AML [15]. Deregulated miR-194-5p contributes to AML tumorigenesis by targeting BCLAF1 [16]. MiR-1303 is a miRNA that has been unveiled to be upregulated and serve a tumorigenic function in several cancers, such as hepatocellular carcinoma [17], neuroblastoma [18] and gastric cancer [19]. However, the role of TPTEP1 or miR-1303 in AML remains unexplored.

In this study, we targeted at investigating the potential role and mechanism of TPTEP1and miR-1303 in AML development.

## Materials and methods

### Bioinformatics analysis

GEPIA 2 (http://gepia2.cancer-pku.cn/#index) was applied to search the expression of TPTEP1 in AML tissues. miRGen v.3 (http://www.microrna.gr/mirgen) was employed to discover possible miRNA that might be modulated by JUN. JASPAR (http://jaspardev.genereg.net/) was employed to predict binding sites of JUN on miR-1303. LncBase 2.0 (https://omictools.com/diana-lncbase-tool) was used to find the possible targeted genes of miR-1303.

### Cell culture and reagent

Acute myeloid leukemia (AML) cell lines (HL60, KG-1, THP-1, NB4, MOLM-14), human embryonic kidney cell (HEK-293 T) and normal human bone marrow stromal HS-5 cells were acquired from Shanghai Cell Bank of the Chinese Academy of Sciences (China). AML cells were cultured with 10% fetal calf serum (FCS; TCS Biosciences, London, UK) in Roswell Park Memorial Institute (RPMI) medium (Invitrogen, Carlsbad, CA, USA), while HEK-293 T and normal cell were maintained in Dulbecco’s modified Eagle’s medium (DMEM; HyClone, Logan, UT, USA) with 10% fetal bovine serum (FBS; Thermo Fisher Scientific, Waltham, MA, USA) and 1% penicillin–streptomycin antibiotics (HyClone). Cells culture was conducted under a 5% CO_2_ atmosphere at 37 ºC. Anisomycin, the activator of JNK, was acquired commercially from Adooq (A11271, Nanjing, China).

### Cell transfection

Confluent HL60 or KG-1 cells in 6-well plates were separately transfected with the pcDNA3.1/TPTEP1 and its corresponding empty vector (Genechem, Shanghai, China) to enhance TPTEP1 expression. The short hairpin RNA (shRNA) of JUN or negative control (sh-NC) (Genechem) was transfected into HL60 and KG-1 cells to knockdown JUN expression. MiR-1303 mimics and miR-1303 inhibitors, together with their respective NC, were produced by Genepharma (Shanghai, China). Cells were transfected for 48 h in line with the protocol of Lipofectamine 2000 Reagent (Invitrogen).

### Quantitative real-time polymerase chain reaction (qRT-PCR)

Total RNA from HL60 or KG-1 cells were isolated via TRIzol reagent (Invitrogen) following the standard instructions. Synthesis of First-strand complementary DNA (cDNA) was accomplished via reverse transcriptase kit (Takara, Tokyo, Japan). SYBR Green PCR Master Mix (Roche, Mannheim, Germany) was utilized to carry out qRT-PCR reactions on an ABI7300 real-time PCR machine (Applied Biosystems, Foster City, CA, USA) and detect the amplified products. Relative gene expression was analyzed by the 2^−ΔΔCt^ method. Glyceraldehyde-3-phosphate dehydrogenase (GAPDH) or U6 small nuclear RNA (snRNA) were used as normalized controls.

### Cell viability assay

After transfection, HL60 or KG-1 cells were seeded in 96-well plates (2000 cells/well; Aladdin, Shanghai). Cell Counting Kit-8 solution (CCK-8; Dojindo, Tokyo, Japan) without FBS was supplemented to indicated wells after cells were cultured for 24, 48, 72 or 96 h. Cell viability was detected via a microplate reader (Bio-Tek Instruments, Hopkinton, MA, USA) at 450 nm.

### 5-Ethynyl-2’-deoxyuridine (EdU) staining

The Click-iT Alexa Fluor 488 Imaging Kit (Invitrogen) was employed as per the user guide. Transfected HL60 or KG-1 cells were washed twice with 3% bovine serum albumin (BSA; Sigma-Aldrich, Burlington, Massachusett, USA) and then treated with EdU-Click reaction-mix (C10637, Thermofisher, USA) at 20 °C for 30 min, followed by rinsing in 3% BSA. Cell nuclei were treated with 4',6-diamidino-2-phenylindole (DAPI) staining in darkness.

### Terminal deoxynucleotidyl transferase-mediated dUTP nick-end labeling (TUNEL) staining

After transfection for 48 h, HL60 or KG-1 cells were rinsed with phosphate-buffered saline (PBS) (Sigma-Aldrich) and maintained in 4% paraformaldehyde (PFA) (Sigma-Aldrich) at room temperature for 30 min. TUNEL assay kit (Roche) was applied in accordance with the specification. The apoptotic cells were observed via a fluorescence microscope (Olympus, Tokyo, Japan). The apoptotic cells were in the green regions, while the cell nuclei were in the blue regions.

### Western blot analysis

Transfected HL60 or KG-1 cells were lysed in radioimmunoprecipitation assay (RIPA) buffer (Beyotime, Shanghai, China). Protein concentration was measured by Pierce BCA Protein Assay kit (Thermo Fisher Scientific, USA) following the protocol provided by the manufacturer. The isolation of protein was achieved with the application of sodium dodecyl sulfate–polyacrylamide gel electrophoresis (SDS-PAGE) (89,888, Thermo Fisher) on 15% gel. Proteins were subsequently shifted onto polyvinylidene fluoride (PVDF) membranes (Millipore, Bedford, MA, USA). Membranes were blocked with 5% non-fat dry milk in Tris-buffered saline Tween-20 (TBST; CST, 9997S, USA) overnight at 4 °C and then incubated with primary antibodies against JNK (1/1500, ab124956, Abcam, Cambridge, USA), p-JNK (1/1000, ab124956, Abcam), c-JUN (1/800, ab31419, Abcam), p-c-JNK (1/1000, ab32385, Abcam) and GAPDH (1/1000, ab8245, Abcam) at room temperature for 3 h. Following primary incubation, membranes were washed with TBST for three times and incubated with secondary antibodies at room temperature for 1 h. The final protein bands were visualized via the enhanced chemiluminescence (ECL) system (Santa Cruz Biotechnology, Santa Cruz, CA, USA).

### Chromatin immunoprecipitation (ChIP) assay

ChIP assays were implemented via ChIP assay kit (Millipore) in tune with the supplier’s requirements. Briefly, HEK-293 T cells were fixed with 1% formaldehyde for 10 min at 37 °C and then sonicated on ice. The sonicated chromatin was subsequently immunoprecipitated with antibodies against c-JUN and immunoglobulin G (IgG) (ab2410, Abcam). The qRT-PCR was applied to detect the precipitated DNA fragments.

### Luciferase reporter assay

MiR-1303 promoter was cloned into the pGL3-basic. The wild-type and mutant binding sequences of JUN in miR-1303 promoter (WT, Site1-Mut, Site2-Mut, Site3-Mut and Site4-Mut) were constructed by Genepharma. TPTEP1-WT and TPTEP1-Mut in pmirGLO luciferase reporter vector were also generated at Genepharma. HEK-293 T cells were co-transfected with the pGL3-miR-1303 promoter and shJUN or its corresponding control and WT or Site1-Mut or Site2-Mut or Site3-Mut or Site4-Mut or the empty vector. Then, HEK-293 T cells were co-transfected separately with the aforementioned reporter vectors and indicated transfection plasmids using Lipofectamine 2000. The Dual Luciferase Reporter Assay System (Promega, Madison, WI, USA) was applied after 48 h as the manufacturer’s directions requested.

### RNA pull down assay

Plasmids miR-1303-WT, miR-1303-Mut and corresponding NC sequences were linearly cut, transcribed and biotinylated for 48 h in vitro with a MAXIscript T7 Transcription Kit (Thermo Fisher Scientific). RNA pull down assay was carried out utilizing Pierce Magnetic RNA–Protein Pull-Down Kit (Thermo Fisher Scientific) in HL60 or KG-1 cell lysates. Cell lysates were cultured with streptavidin-coated magnetic beads (Ambion, Austin, TX, USA) for 48 h. The biotin-coupled RNA (Bio-RNA) complex was pulled down and subjected to qRT-PCR after purification.

### Argonaute 2 (Ago2)-RNA binding protein immunoprecipitation (RIP) assay

Magna RIP™ RNA Binding Protein Immunoprecipitation Kit (Millipore) was applied to perform Ago2-RIP assay based on the protocol. Cells were reaped at a confluence of 80–90% and lysed in RIP lysis buffer with magnetic bead conjugated and anti-Ago2 antibody (Millipore) or IgG overnight at 4 °C. After digesting with proteinase K (Absin, Shanghai, China), the immunoprecipitated RNA was purified and analyzed by qRT-PCR.

### Statistical analysis

All Data were presented as mean ± standard deviation (SD) from at least three independent experiments. A value of P < 0.05 was considered statistically significant. Differences analysis throughout this study was assessed by Student’s t-test. One-way or two-way analysis of variance (ANOVA) was involved in difference analysis and Dunnett’s test or Tukey served as back testing methods. All analysis was carried out via SPSS 23.0.

## Results

### TPTEP1 plays a tumor suppressive part in AML

**Fig. 1:**
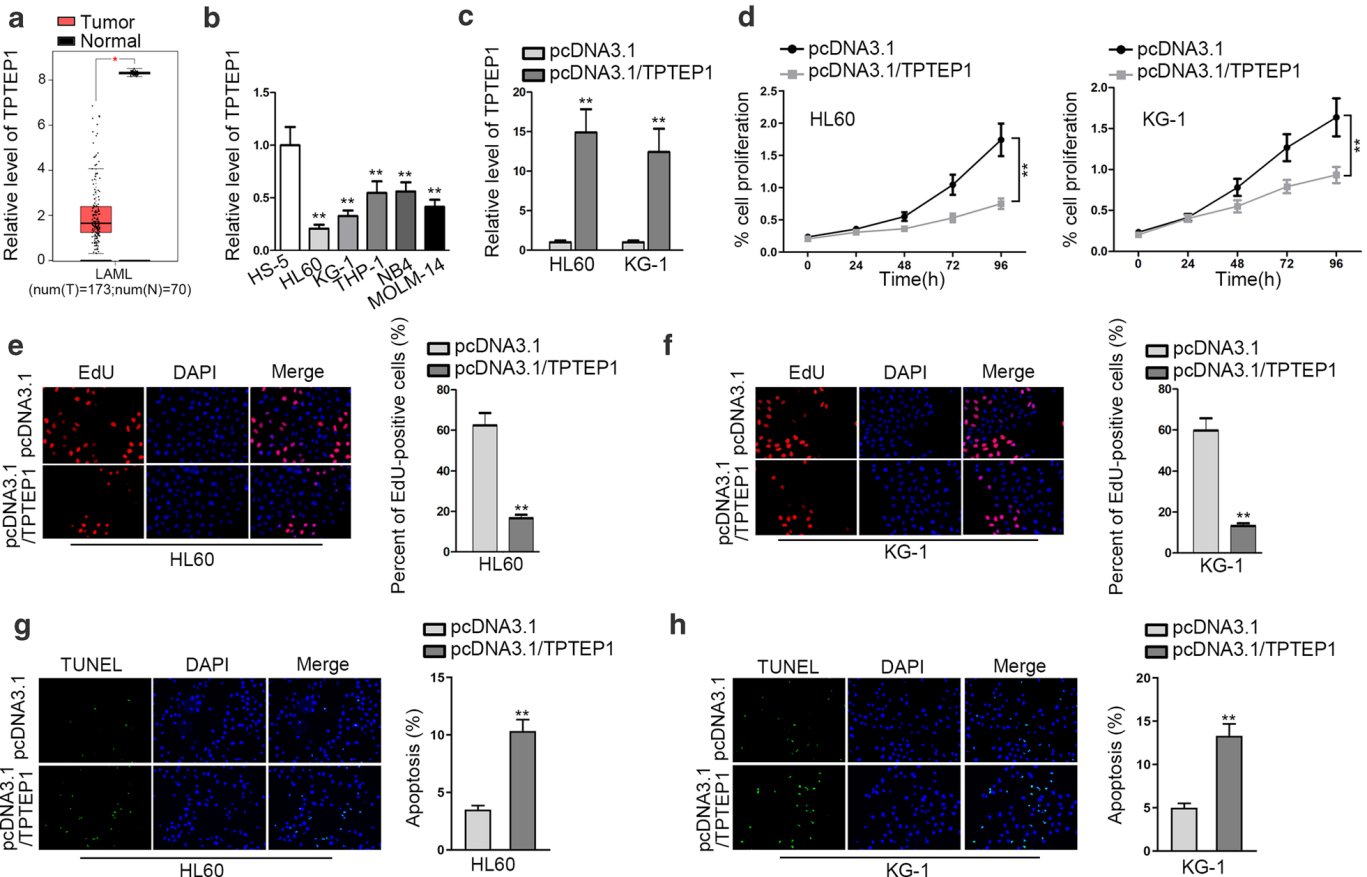
TPTEP1 impeded the proliferation of AML cells. **a** GEPIA 2 database suggested Transmembrane phosphatase with tensin homology pseudogene 1 (TPTEP1) was decreased in acute myeloid leukemia (AML) samples compared with the normal controls. **b** Relative expression of TPTEP1 in AML cell lines and the normal control HS-5 cells was analyzed by quantitative real-time polymerase chain reaction (qRT-PCR). **c** QRT-PCR result of TPTEP1 expression in HL60 and KG-1 cells under the transfection of pcDNA3.1 or pcDNA3.1/TPTEP1. **d** Cell proliferation was evaluated through cell counting kit-8 (CCK-8) assay. **e, f** The proliferation of HL60 and KG-1 cells under TPTEP1 upregulation or not was determined by 5-Ethynyl-2’-deoxyuridine (EdU) assays. (G-H) TUNEL assay was conducted to assess cell apoptosis rate of above cells. Data were presented as mean ± standard deviation (SD) (error bar). *P < 0.05, **P < 0.01

To fathom out the function of TPTEP1 in AML, we first searched for the expression of TPTEP1 in GEPIA 2. The data from GEPIA 2 showed that TPTEP1 was low-expressed in 173 AML samples in comparison to 70 normal samples (Fig. 1a). Also, we found that TPTEP1 expression was remarkably decreased in all the five AML cell lines, especially in HL60 and KG-1 cells, relative to the normal control HS-5 cells (Fig. 1b). Therefore, we overexpressed TPTEP1 in these two cells to determine the precise function of TPTEP1 in AML cells. As indicated by the qRT-PCR outcome, the level of TPTEP1 was extremely upregulated in both HL60 and KG-1 cells in response to pcDNA3.1/TPTEP1 transfection (Fig. 1c). Additionally, cell proliferation was markedly restrained in HL60 and KG-1 cells under TPTEP1 overexpression (Fig. 1d). Consistently, elevated expression of TPTEP1 mitigated the percentage of EdU-positive HL60 and KG-1 cells (Fig. 1e, f). In contrast, the apoptosis rate was overtly encouraged in TPTEP1-overexpressed HL60 and KG-1 cells (Fig. 1g, h). Therefore, we concluded that TPTEP1 suppressed cell proliferation but induced cell apoptosis in AML.

### TPTEP1 inhibits JNK/c-JUN signaling in the leukemogenesis of AML

**Fig. 2:**
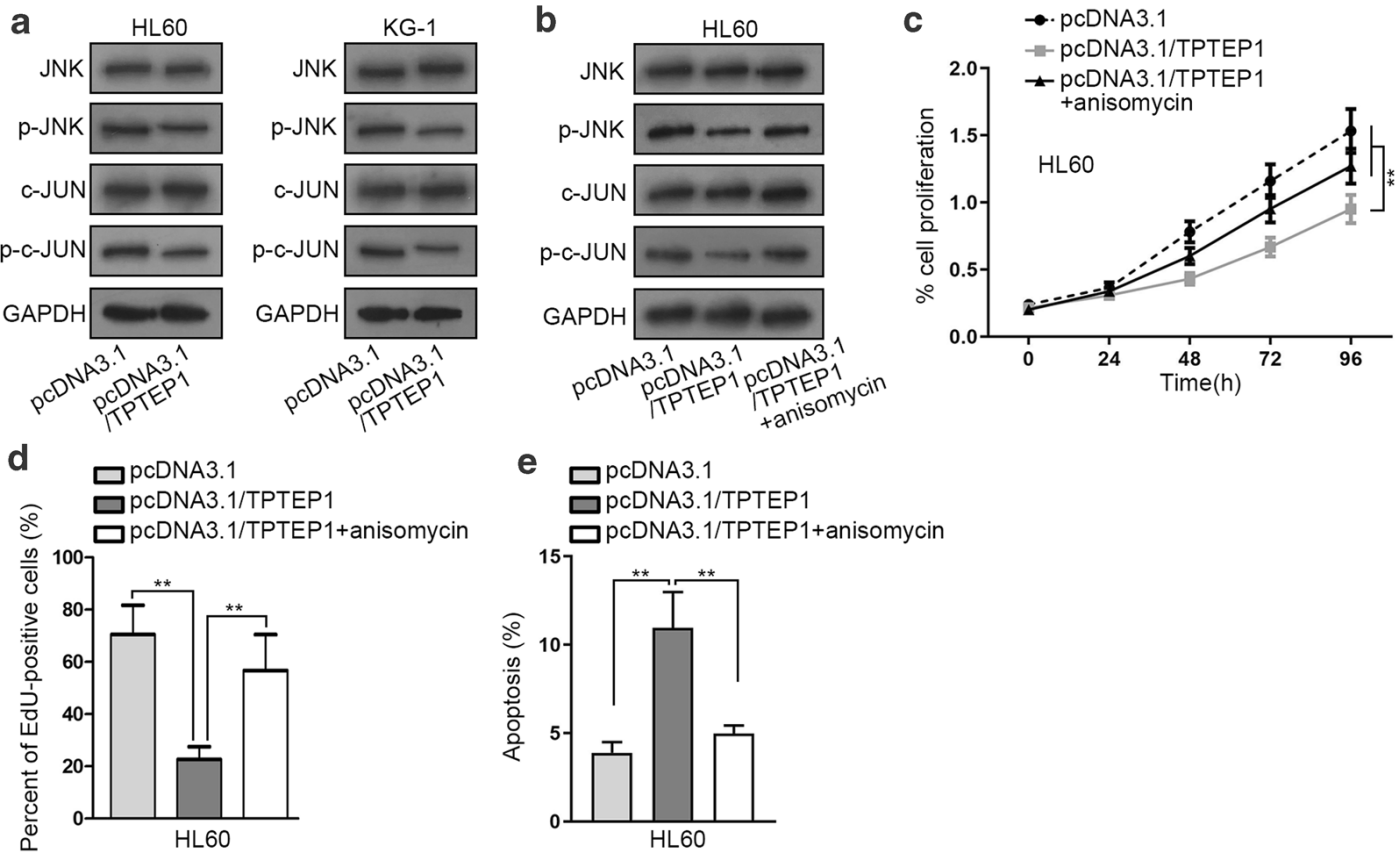
TPTEP1 restrained AML cell growth through inactivating JNK/c-JUN signaling. **a,** **b** The level of proteins involved in c-Jun N-terminal kinase (JNK)/c-JUN pathway in indicated AML cells was presented by means of western blot. **c–e** The proliferative and apoptotic capabilities of HL60 cells under different transfections were estimated by CCK-8, EdU, and TUNEL assays. Data were presented as mean ± SD (error bar). **P < 0.01

It is reported certain gene could activate JNK signaling in multiple cancers including leukemia [20]. In this study, we wondered whether TPTEP1 also had an effect on this pathway. The western blot results suggested that the phosphorylation of JNK and c-JUN was hindered in both HL60 and KG-1 cells under TPTEP1 upregulation (Fig. 2a), indicating the negative regulation of TPTEP1 on JNK/c-JUN signaling pathway. Anisomycin is widely used as an agonist for in studies investigating the JNK signaling pathways [21]. To further confirm whether TPTEP1 affected AML cell growth through JNK/c-JUN pathway, the rescue assays were conducted in HL60 cells employing anisomycin. As proved in Fig. 2b, the levels of p-JNK and p–c-JUN that were lessened by TPTEP1 overexpression were recovered upon JNK activation. Moreover, the impeded proliferation of TPTEP1-upregulated AML cells was normalized after activating JNK/c-JUN signaling (Fig. 2c, d), whereas TPTEP1 overexpression-enhanced apoptosis of HL60 cells was attenuated in response to anisomycin treatment (Fig. 2e). Collectively, TPTEP1 controlled AML cell growth through inactivating JNK/c-JUN pathway.

### JUN positively regulates miR-1303 in AML cells at transcriptional level

**Fig. 3:**
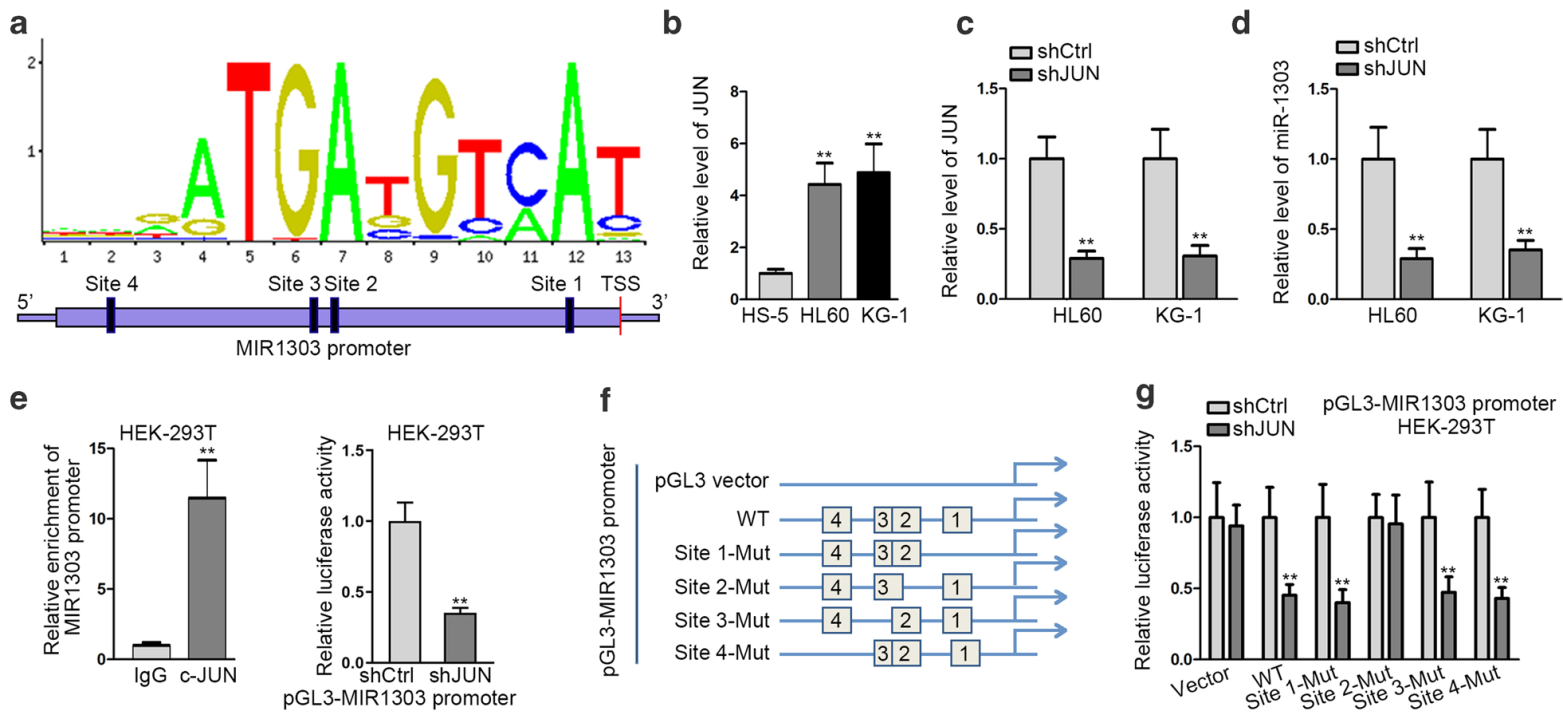
JUN positively regulated miR-1303 in AML cells at transcriptional level. **a** JASPAR predicted that there existed four possible binding sites of c-JUN and microRNA(miR)-1303 promoter. **b** The expression level of JUN in HL60, KG-1 and normal control HS-5 cells was examined with the help of qRT-PCR. **c, d** The expression of JUN and miR-1303 in HL60 and KG-1 cells with or without JUN silence was determined through qRT-PCR. **e** The effect of c-JUN on miR-1303 transcription was assessed by chromatin immunoprecipitation (ChIP) and luciferase reporter assays. **f–g** Luciferase reporter assay was implemented to verify the precise binding site of c-JUN to miR-1303 promoter. Data were presented as mean ± SD (error bar). **P < 0.01

Given that JUN is a transcription factor that usually functions as an oncogene through modulating its target genes, we were curious about the downstream target of JUN that was involved in TPTEP1-regulated AML cells. As predicted by miRGen v.3, it was largely suggested that JUN was a potential regulator of miR-1303. MiR-1303 is reported to be upregulated and served a pro-tumor part in several cancers. Meanwhile, the JASPAR predicted that there were 4 putative binding sites of JUN on the region of miR-1303 promoter (Fig. 3a). Meanwhile, we discovered the expression of JUN was apparently stimulated in HL60 and KG-1 cells compared with that in the normal control HS-5 cells (Fig. 3b). Importantly, it turned out that miR-1303 expression was also reduced along with the decreased JUN level in AML cells (Fig. 3c, d). Also, miR-1303 promoter was revealed to be greatly enriched in the complex immunoprecipitated by anti-c-JUN, while the luciferase activity of miR-1303 promoter sharply lessened in the context of JUN knockdown (Fig. 3e), suggesting JUN was a positive regulator of miR-1303 transcription. Furthermore, to confirm the precise binding sites of JUN on miR-1303 promoter, we performed luciferase reporter assays in HEK-293 T cells with the transfection of pGL3 plasmids containing different sequences of miR-1303 promoter (Fig. 3f). As a consequence, we confirmed that the attenuation of c-JUN on miR-1303 transcription was only hindered when mutating the sequence of site 2 (Fig. 3g), suggesting the specific binding sequence of c-JUN and miR-1303 promoter was at site 2. Altogether, we uncovered that miR-1303 was transcriptionally enhanced by c-JUN in AML cells.

### TPTEP1 directly interacts with miR-1303 in AML cells

**Fig. 4:**
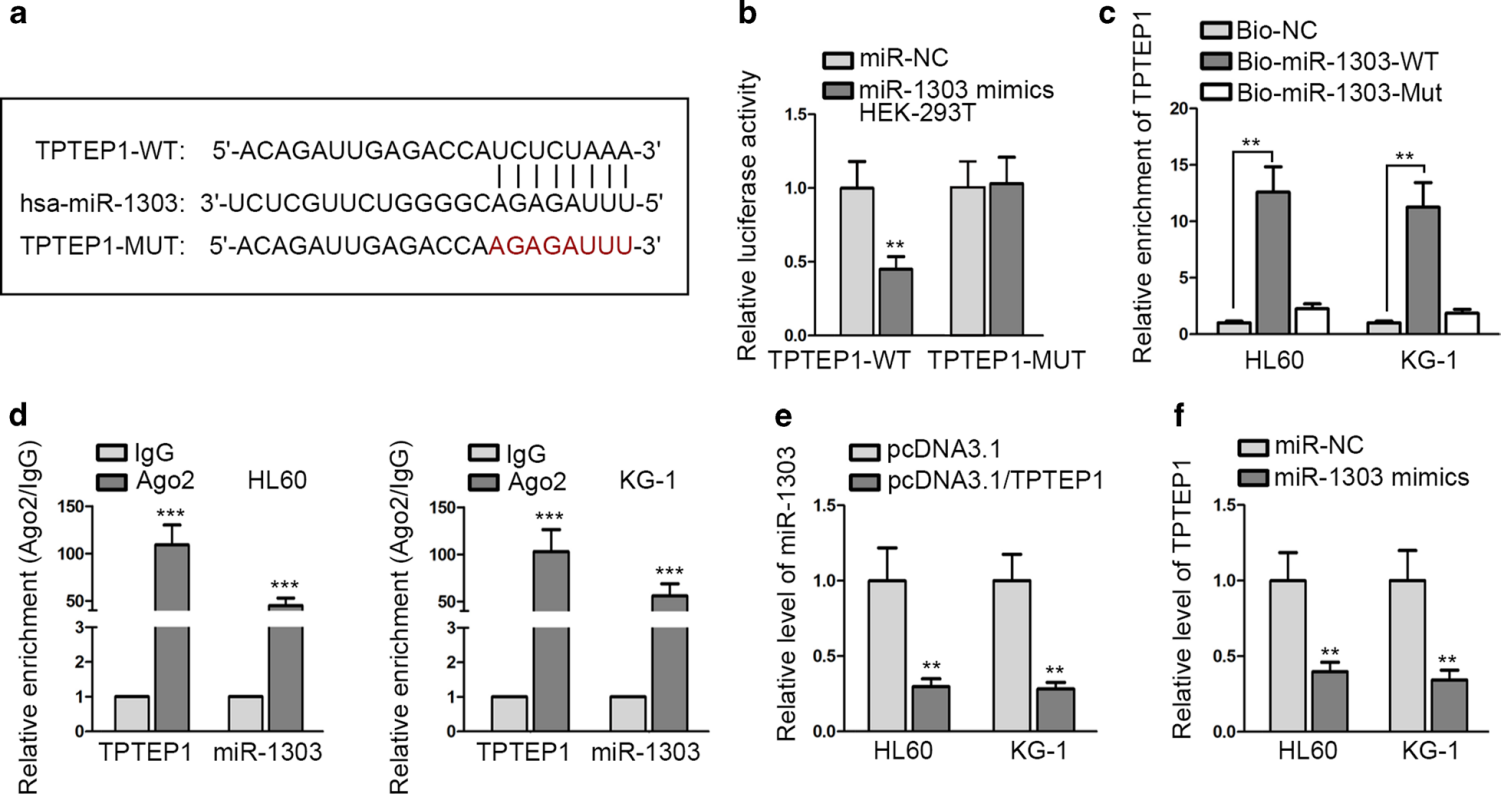
TPTEP1 interacted with miR-1303 in AML cells. **a** Corresponding sequences were exhibited. **b–d** The interaction between TPTEP1 and miR-1303 was verified in luciferase reporter assay (**b**), RNA pull down assay (**c**) and RIP assay (**d**). **e** The level of miR-1303 in HL60 and KG-1 cells under TPTEP1 overexpression was evaluated by qRT-PCR. **f** QRT-PCR result of TPTEP1 expression in AML cells under miR-1303 upregulation. Data were presented as mean ± SD (error bar). **P < 0.01, ***P < 0.001

Recently, lncRNAs have been demonstrated to sponge miRNAs and therefore facilitate tumor development in numerous cancers, including AML. For example, previous research manifested that lncRNA KCNQ1OT1 contributed to the progression of AML by sponging miR-193a-3p [22]. In this regard, we wanted to know whether there was an interaction between TPTEP1 and miR-1303 in AML cells. According to the result of bioinformatics analysis, it was suggested that there was a possible affinity of miR-1303 and TPTEP1 (Fig. 4a). In addition, ectopic expression of miR-1303 resulted in sharply weakened luciferase activity of TPTEP1-WT not that of TPTEP1-Mut (Fig. 4b). Moreover, the RNA pull down result unveiled that TPTEP1 was distinctly enriched only by Bio-miR-1303-WT, but never by Bio-NC or Bio-miR-1303-Mut (Fig. 4c). Moreover, we further certified that the combination relation between TPTEP1 and miR-1303 in AML cells happened in RNA-induced silencing complex (RISC) (Fig. 4d). Additionally, we revealed that the level of miR-1303 was strikingly diminished in TPTEP1-overexpressed HL60 and KG-1 cells, whereas the expression of TPTEP1 was also dramatically abated in above two AML cells under miR-1303 upregulation (Fig. 4e, f). By and large, these data elucidated that TPTEP1 and miR-1303 was mutually repressed by each other in AML cells.

### Upregulation of miR-1303 countervails the inhibition of TPTEP1 upregulation on AML cell malignant behaviors

**Fig. 5:**
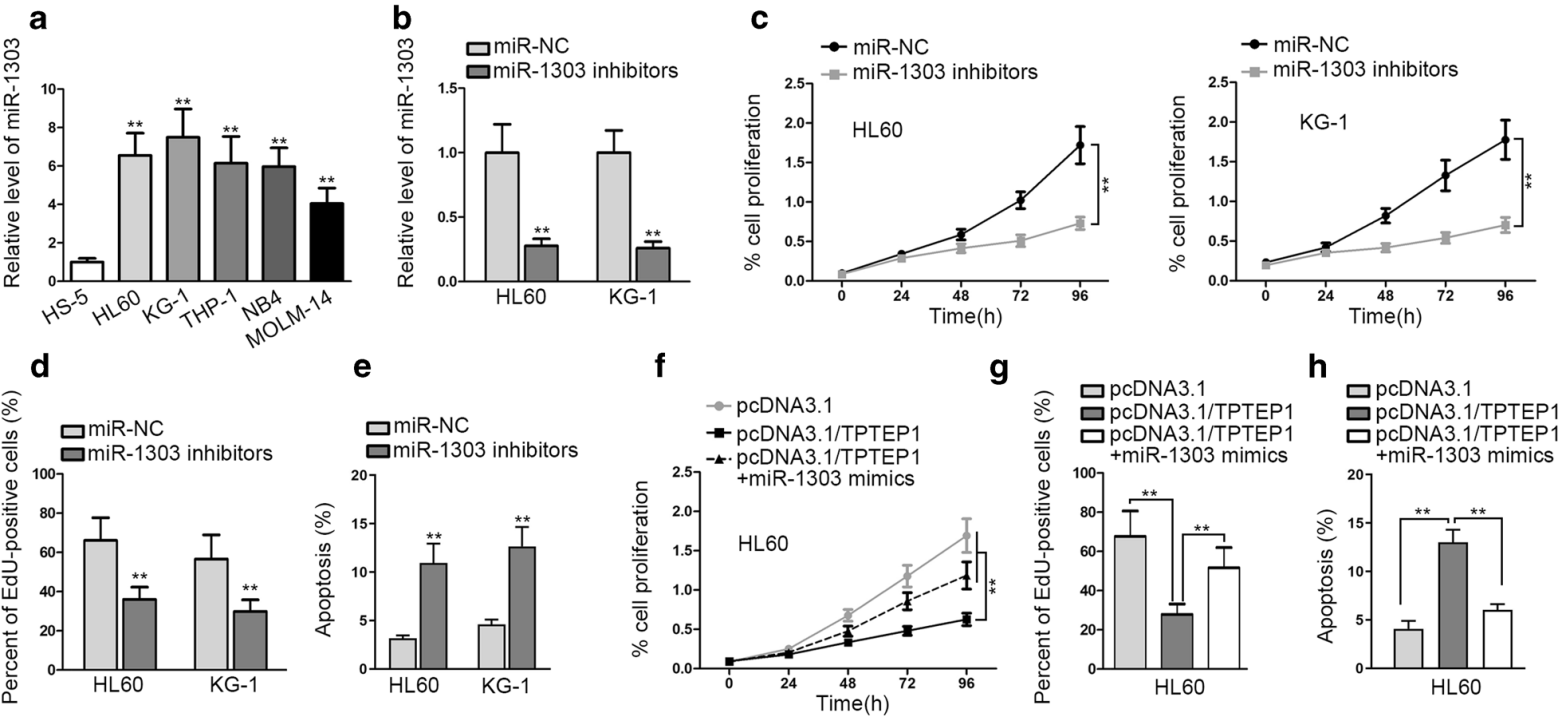
MiR-1303 was an oncogene in AML cells and its upregulation offset the inhibition of TPTEP1 overexpression on AML cell growth. **a** QRT-PCR result of miR-1303 in AML cells and normal HS-5 cells. **b** The expression of miR-1303 in HL60 and KG-1 cells after transfection with miR-negative control (NC) or miR-1303 mimics was quantified with the assistance of qRT-PCR. **c–h** CCK-8, EdU and TUNEL assays were carried out to estimate proliferation and apoptosis of HL60 cells under diverse conditions, as appropriate. Data were presented as mean ± SD (error bar). **P < 0.01

In this basis, we suspected that miR-1303 might be one of the possible molecules that participated in TPTEP1-affected AML cell growth. To certify this assertion, we first investigated the exact role of miR-1303 in AML cells. As displayed in Fig. 5a, miR-1303 was remarkably elevated in AML cells relative to normal HS-5 cells. Intriguingly, inhibition of miR-1303 led to restrained proliferation as well as promoted apoptosis of both HL60 and KG-1 cells (Fig. 5b–e), suggesting miR-1303 was a tumor-promoter in AML cell malignant process. Moreover, we further illustrated that the suppressed proliferative ability of HL60 cells with TPTEP1 upregulation was reversed in response to ectopic expression of miR-1303 (Fig. 5f–g). By contrast, TPTEP1 overexpression-encouraged apoptosis noticeably declined in AML cells facing miR-1303 upregulation (Fig. 5h). All in all, miR-1303 participated in the growth of TPTEP1-regulated AML cells.

## Discussion

TPTEP1 is a lncRNA that has been previously recognized as a tumor suppressor in hepatocellular carcinoma [13], while miR-1303 has been revealed to be upregulated and accelerate tumor development in several human cancers, such as hepatocellular carcinoma [17], neuroblastoma [18] and gastric cancer [19]. In this study, we uncovered for the first time that TPTEP1 was an anti-tumor lncRNA and that miR-1303 was a facilitator in AML.

Moreover, we unveiled that TPTEP1 confined AML cell proliferation through inactivating JNK/c-JUN signaling, a well-known oncogenic pathway verified in a variety of cancers [23–25], including AML [26]. C-JUN is a transcription factor that is commonly upregulated in AML [27], and its activation by JNKs leads to increased transcription of a wide range of the target genes [28]. Presently, it was indicated that the activation of JNK/c-JUN pathway was repressed by TPTEP1 upregulation, while c-JUN was a positive regulator on miR-1303 transcription, which explained why miR-1303 was controlled by TPTEP1 in AML.

In turn, we also evaluated whether there was an interaction of miR-1303 with TPTEP1 in AML, as the function of lncRNAs as a miRNA decoy has been demonstrated recently [29]. Fortunately, the LncBase 2.0 predicated that there was a potential interaction between TPTEP1 and miR-1303, and the interaction between them was further validated in the present study. Moreover, it was indicated that TPTEP1 was also negatively regulated by miR-1303, which was owing to the RNA knockdown induced by miR-1303-assembled RISC [30, 31]. Furthermore, it was certified that miR-1303 was implicated in TPTEP1-suppressed AML development.

## Conclusion

In conclusion, our study disclosed a TPTEP1/JNK/c-JUN/miR-1303 signaling in the development of AML. Hence, two novel molecules, TPTEP1 and miR-1303, were evidenced as two promising therapeutic targets for AML treatment, although their clinical value should be further strengthened in future studies.

## Data Availability

Research data not shared.

## Abbreviations

BCA: Bicinchoninic acid
ChIP: Chromatin immunoprecipitation
DAPI: 4',6-Diamidino-2-phenylindole
ECL: Enhanced chemiluminescence
EdU: 5-Ethynyl-2’-deoxyuridine
GAPDH: Glyceraldehyde-3-phosphate dehydrogenase
JNK: C-Jun N-terminal kinase
AML: Acute myeloid leukemia
NC: Negative control
PBS: Phosphate buffer saline
PCR: Polymerase chain reaction
PFA: Paraformaldehyde
PVDF: Polyvinylidene fluoride
RIP: RNA binding protein immunoprecipitation
RIPA: Radio immunoprecipitation assay
RPMI: Roswell Park Memorial Institute
SDS-PAGE: Sodium dodecyl sulfate–polyacrylamide gel electrophoresis
TBST: Tris-buffered saline Tween-20
TPTEP1: Transmembrane phosphatase with tensin homology pseudogene 1
TUNEL: Terminal deoxynucleotidyl transferase-mediated dUTP nick-end labeling
